# Molecular detection of bee pathogens in honey from various botanical origins

**DOI:** 10.1371/journal.pone.0336324

**Published:** 2025-12-10

**Authors:** Rossella Tiritelli, Gian Luigi Marcazzan, Cecilia Costa, Antonio Nanetti, Giovanni Cilia

**Affiliations:** CREA Research Centre for Agriculture and Environment (CREA-AA), Bologna, Italy; University of Murcia: Universidad de Murcia, SPAIN

## Abstract

Honey bees play a crucial role in pollination and global food security, yet their populations are declining due to various environmental stressors, including pathogenic infections. Recently, molecular research in honey has been proposed as a powerful, non-invasive tool for detecting and monitoring honey bee pathogens and parasites. This study analysed 679 honey samples from all Italian regions to detect the presence of 8 pathogens (DWV, CBPV, ABPV, BQCV, KBV, *Nosema ceranae*, *Crithidia mellificae*, *Lotmaria passim*) using qPCR assays. Overall, 97.5% of the honey samples tested positive for at least one pathogen, with the most prevalent being DWV (81.7%), *N. ceranae* (56.1%), and CBPV (56.0%). None of the samples tested positive for KBV or *C. mellificae*. Statistical analyses revealed significant variations in pathogen prevalence and copy number depending on the honey type, geographic origin and correlations among different pathogens. Additionally, co-presence was common, with over 77% of honey samples containing multiple pathogens. These findings support honey analysis as an effective and valid method for pathogen surveillance in honey bee populations. By providing valuable insights into disease dynamics, this approach could enhance epidemiological monitoring and contribute to improved honey bee health management strategies.

## Introduction

Honey bees (*Apis mellifera*) are the most important commercial pollinators worldwide [1]. Their role is crucial in ensuring the reproduction of a vast range of plant species, enhancing agricultural productivity, and supporting global food security [2–4]. However, in recent decades, honey bee populations have experienced phenomena of collapse and population declines due to various environmental stressors, including habitat loss, pesticide exposure, climate change, and, notably, pathogen infections [5,6]. Among these threats, the spread of pathogens poses a serious risk to colony health and survival, with viruses, bacteria, fungi, trypanosomatids and parasites contributing to colony losses [7,8].

Traditional pathogen monitoring in honey bee colonies typically involves direct sampling of individual bees, a process that can be both logistically demanding and invasive, requiring the collection of numerous specimens [9]. However, pathogens can also spread through hive products such as honey, pollen, and royal jelly, facilitating their transmission to previously uninfected colonies [10,11]. With the increasing international trade of hive products and honey bee colonies [12], the risk of pathogen dissemination across geographic regions has intensified, leading to new outbreaks in areas where these pathogens had not been previously recorded [13–15]. Recent advancements in molecular techniques have introduced environmental DNA (eDNA) and environmental RNA (eRNA) as a promising tool to use honey as a bioindicator [9,16–18]. The eDNA and eRNA refer to genetic material extracted from environmental samples (e.g., water, soil, air or biological matrices such as faeces, blood etc) rather than directly from an organism, offering a powerful and precise method for identifying cryptic, elusive, and invasive pathogens [19,20].

Honey, primarily composed of sugars and minor components, has long been used to assess environmental contaminants and pollutants [21,22]. Similarly, it serves as a valuable environmental source of eDNA and eRNA, capturing traces of organisms present in the ecological niche where it was produced, including honey bee parasites and pathogens [23–29]. Recent studies have demonstrated the utility of honey in sequencing the mitochondrial DNA of *Varroa destructor* [23,30], a parasitic mite that infests honey bee colonies. Additionally, honey has been proposed as a tool for detecting hive infections caused by bacteria [26–30], fungi [24,30], trypanosomatids [25,30], and other parasites [31]. Given its potential for non-invasive and large-scale monitoring, honey analysis provides an effective approach to assessing pathogen prevalence and distribution across extensive geographical areas [25,30–32].

This study aims to evaluate honey as a non-invasive surveillance matrix for bee pathogens by applying quantitative PCR to detect and quantify eight microorganisms —*Nosema ceranae* (Microsporidia:Nosematidae), *Lotmaria passim* and *Crithidia mellificae* (Kinetoplastea: Trypanosomatidae), deformed wing virus (DWV), black queen cell virus (BQCV), chronic bee paralysis virus (CBPV), acute bee paralysis virus (ABPV) and Kashmir bee virus (KBV)— in honey samples collected across all 20 Italian regions and spanning both monofloral and multifloral botanical origins. Estimates of pathogen prevalence, loads, and co-occurrence were obtained, and variation by honey type, region, and broader geographic area was assessed, with the overarching objective of establishing an epidemiological baseline for colony health and validating honey-based eDNA/eRNA analysis as a scalable tool for risk assessment and apiary health management.

## Materials and methods

A total of 679 honey samples from all of the 20 regions of Italy were collected (Table 1), provided directly by beekeepers participating in the MEDIBEES project (N = 65) and by Osservatorio Nazionale Miele (N = 614) through the “Tre Gocce d’Oro 2023” honey awards. Among the samples investigated, 475 honeys were monofloral, while 204 were multifloral. The botanical origins of the investigated honey were declared by the producer’s beekeeper and confirmed by a certified sensory analysis panel. S1 Table reports the list of samples, including their botanical origin and production region. No honey was heat-treated before and after sampling.

**Table 1:** Total number of samples collected from all 20 regions of Italy.

Italian region	N° honey samples
Abruzzo	45
Aosta Valley	16
Apulia	60
Basilicata	32
Calabria	18
Campania	36
Emilia-Romagna	29
Friuli-Venezia Giulia	17
Lazio	22
Liguria	15
Lombardy	64
Marche	20
Molise	27
Piedmont	54
Sardinia	87
Sicily	29
Trentino-Alto Adige	24
Tuscany	30
Umbria	14
Veneto	40

### Extraction of nucleic acids

For each sample, 50 g of honey were homogenized with 150 mL of ultrapure water, vortexed, and incubated at 40 °C for 30 minutes. The suspension was then divided into five separate 50 mL tubes and centrifuged at 5000 × g for 40 minutes at room temperature, after which the supernatant was discarded. The resulting pellet was resuspended in 0.5 mL of ultrapure RNase- and DNase-free water and the contents of the five tubes were combined into one before undergoing a second centrifugation at 5000 × g for 30 minutes at room temperature. Finally, the supernatant was discarded, and the pellet was resuspended in 1 mL of ultrapure RNase- and DNase-free water.

Each homogenate was crushed for 3 min at 30 Hz TissueLyser II (Qiagen, Hilden, Germany), as previously reported [33]. The suspensions were separated into two aliquots from which DNA and RNA were extracted separately. The extraction of the DNA and RNA was performed using a Quick DNA Microprep Plus Kit (Zymo Research, Irvine, CA, USA) and a Quick RNA Microprep Plus Kit (Zymo Research), respectively [34,35]. The extracted nucleic acids were eluted in 100 µl of DNAase-RNase-free water and kept at −80°C until the qPCR analysis.

### Quantitative Real-Time PCR assays

A quantitative Real-Time PCR (qPCR) analysis was performed to determine the copy number of each pathogen in the samples using the extracted DNA, used for *Nosema ceranae*, *Lotmaria passim* and *Crithidia mellificae*, and RNA, used for DWV, BQCV, CBPV, ABPV and KBV. A total reaction volume of 25 µl was produced for each target gene using SYBR™ green assays with forward and reverse primers and nucleic acid extract adding 5 µl of extracted DNA or RNA. The SYBR PowerUp™ SYBR™ Green Master Mix (ThermoFisher, Waltham, MA, USA) and the one-step Power SYBR™ Green Cells-to-CT™ Kit (ThermoFisher Scientific) were used for the DNA and RNA, respectively. The primers used for the qPCRs are reported in Table 2. The qPCRs were carried out using a QuantStudio™ 3 Real-Time PCR System (ThermoFisher Scientific), according to the protocols for each gene sequence [36–39]. The bee housekeeping gene *Ribosomal Protein L32* (*RPL32*) was used as an internal control to evaluate the extraction quality [40].

**Table 2:** List of primers used to detect the investigated microsporidian, trypanosomatids and viruses.

Target	Primer name	Sequence (5’-3’)	Reference
*Nosema ceranae*	Hsp70_F Hsp70_R	GGGATTACAAGTGCTTAGAGTGATT TGTCAAGCCCATAAGCAAGTG	[37]
*Lotmaria passim*	TOPII_F TOPII_R	GGCCATGGAAATACTCGAGTCT ACCTTGCCTTCCTTCTTGAGATT	[36]
*Crithidia mellificae*	Cytb_F Cytb_R	TTTTGCCATGCACTATGATGTCT AACCTATTACAGGCACAGTTGCTAAA	
DWV	DWV8450_F DWV8953_F	TGGCATGCCTTGTTCACCGT CGTGCAGCTCGATAGGATGCCA	[38]
BQCV	BQCV9195_F BQCV8265_R	GGTGCGGGAGATGATATGGA GCCGTCTGAGATGCATGAATAC	[39]
CBPV	CPV304_F CPV371_R	TCTGGCTCTGTCTTCGCAAA GATACCGTCGTCACCCTCATG	
ABPV	APV95_F APV159_R	TCCTATATCGACGACGAAAGACAA GCGCTTTAATTCCATCCAATTGA	
KBV	KBV83_F KBV161_R	ACCAGGAAGTATTCCCATGGTAAG TGGAGCTATGGTTCCGTTCAG	
RPL32	RPL32_F RPL32_R	AGTAAATTAAAGAGAAACTGGCGTAA TAAAACTTCCAGTTCCTTGACATTAT	[40]

DNA and RNA previously extracted from infected honey bees, available in the CREA-AA lab from other investigations, were used as positive controls for each pathogen investigated, while ultrapure RNase- and DNase-free water worked as the negative control. All analyses were performed in duplicate. The upper cycle threshold (Ct) of 35 was applied for positive pathogen reactions to minimize the risk of false positives.

For each target gene, a standard curve was created by amplifying serially diluted recombinant plasmids containing the pathogen-specific DNA and RNA fragment from 1 × 10^1^ to 1 × 10^9^ copies in a qPCR assay on QuantStudio TM 3 Real-Time PCR System (ThermoFisher Scientific), as previously reported [41,42].

DWV, deformed wing virus; BQCV, black queen cell virus; CBPV, chronic bee paralysis virus; ABPV, acute bee paralysis virus; KBV, Kashmir bee virus; RPL32: Ribosomal Protein L32.

### Statistical analysis

Pathogens’ prevalence values (calculated as the number of positive samples out of the total samples collected) and copy number values (determined as the log10 transformed averages of the results obtained from two technical replicates) were used for the statistical analysis.

A principal component analysis (PCA) was performed to explore similarities and potential clustering of pathogen copy numbers according to honey types, regions, and geographical classifications (northwestern, northeastern, central, southern, and insular). Heatmaps were created to visualize the prevalence and copy number of pathogens across the investigated variables (honey type, region, and geographical classification). Additionally, an explorative analysis was conducted using Spearman’s correlation to assess relationships between pathogen copy numbers.

The Shapiro-Wilk test was employed to test the normality of data distribution. Since the normality test failed, a non-parametric approach was adopted. The effects of honey types, regions, and geographical classifications on the prevalence and copy number of each pathogen were analysed using the Kruskal-Wallis test. For significant results, *post-hoc* pairwise comparisons were conducted using the Dunn test.

All statistical tests were performed at a significance level of α = 0.05. Pathogens that were not detected in any of the samples were excluded from the analysis.

All statistical calculations were performed using RStudio (version 4.4.2), utilizing the *factoextra*, *FactoMineR*, *Hmisc*, *corrplot*, *corrgram*, *pheatmap*, *FSA* and *ggplot2* packages [43–49].

## Results

None of the samples tested positive for KBV or the trypanosomatid *C. mellificae*. The most frequently detected pathogens were DWV (81.71%), *N. ceranae* (56.11%), and CBPV (55.96%), followed by BQCV (13.11%), *L. passim* (5.15%), and ABPV (4.27%), which were less commonly present (Table 3). Regarding copy number, the highest average number of pathogen copies was found for DWV and CBPV (1.06 × 10^11^ and 7.87 × 10^8^, respectively), followed by *N. ceranae* (5.54 × 10^6^), *L. passim* (5.54 × 10^6^), ABPV (1.48 × 10^6^), and BQCV (8.35 × 10^3^) (Table 3).

**Table 3:** Pathogen prevalence and mean copy number (± standard deviation).

Pathogen	N° of positive samples	Prevalence	Mean copy number	Standard deviation
DWV	555	81.74%	1.06 × 10^11^	1.28 × 10^12^
CBPV	380	55.96%	7.87 × 10^8^	1.30 × 10^10^
ABPV	29	4.27%	1.48 × 10^6^	2.44 × 10^7^
BQCV	89	13.11%	8.35 × 10^3^	1.98 × 10^5^
KBV	0	–	–	–
*N. ceranae*	381	56.11%	5.54 × 10^6^	6.47 × 10^7^
*C. mellificae*	0	–	–	–
*L. passim*	35	5.15%	5.54 × 10^6^	1.29 × 10^8^

**Fig 1:**
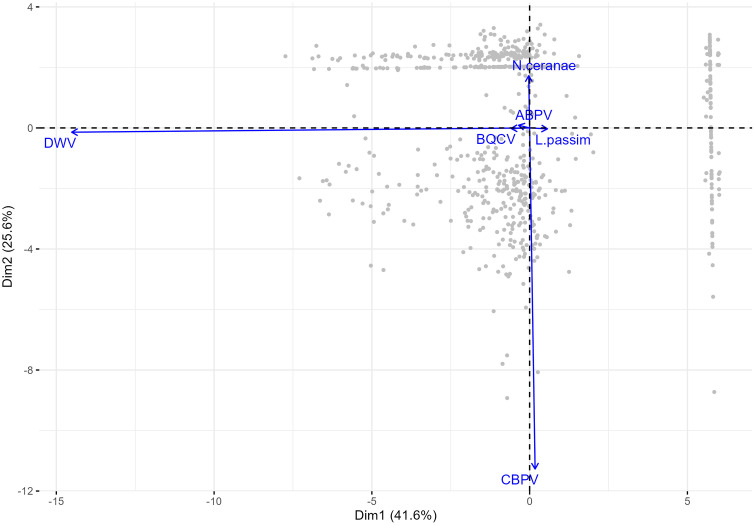
Graphical interpretation (biplot) of principal component analysis (PCA) on pathogen copy numbers. Each grey point represents the pathogen copy number for each analysed sample.

In the PCA, the variation of pathogen copy number is primarily explained by two components. The first component (Dim1), which accounts for 41.6% of the variability, was negatively correlated with DWV (Fig 1). Additionally, the second component (Dim2), which explains 25.6% of the variability, was negatively correlated with CBPV (Fig 1). The principal component analysis did not reveal any clustering associated with honey type, region, or geographical classification. S2 Table provides details on the relationship between Principal Components (PC1 and PC2) and pathogen copy number variables.

A positive correlation was detected between the copy number of DWV and ABPV, as well as DWV and BQCV. S3 Table reports p-value correlations between pathogens.

The botanical origin of honey significantly influenced the prevalence of DWV (Kruskal-Wallis, χ^2^ = 78.60; df = 36; p < 0.001), CBPV (Kruskal-Wallis, χ^2^ = 78.85; df = 36; p < 0.001), and ABPV (Kruskal-Wallis, χ^2^ = 63.56; df = 3; p < 0.01) (S1 Fig). DWV prevalence in ivy (*Hedera helix*) honey was statistically lower compared to strawberry tree (*Arbutus unedo*), French honeysuckle (*Sulla coronaria*), rapeseed (*Brassica napus*), multifloral, linden (*Tilia* L.), eucalyptus (*Eucalyptus* L.), heather (*Erica* L.), chestnut (*Castanea* L.), citrus (*Citrus* L.), acacia (*Robinia pseudoacacia*), and asphodel (*Asphodelus* L.) honeys (S4 Table ). Lavender (*Lavandula* L.) honey showed a statistically lower prevalence of CBPV compared to strawberry tree honey, while citrus honey exhibited a statistically higher prevalence of CBPV compared to lavender and eucalyptus honeys (S4 Table ). ABPV was detected in significantly higher numbers in teucrium (*Teucrium* L.) and reynoutria (*Reynoutria japonica*) honey samples compared to 29 other honey types (S4 Table ). For all other pathogens, no statistically significant differences were observed based on botanical origin. The effect of region or geographical area of honey production on pathogen prevalence was not statistically significant, except for *L. passim*. The region of honey origin influenced the prevalence of this trypanosomatid (Kruskal-Wallis, χ^2^ = 39.609; df = 19; p < 0.01), with higher numbers observed in honey from Tuscany compared to Abruzzo (S1 Fig and S4 Table).

**Fig 2:**
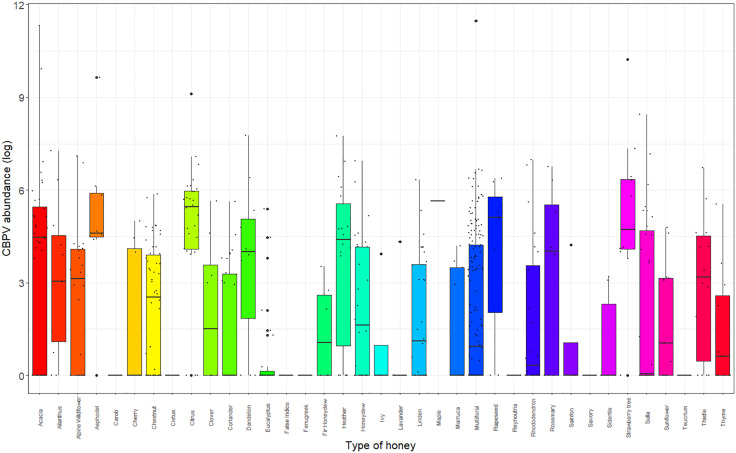
Boxplot of CBPV copy number in different types of honey. Copy numbers are represented as decimal logarithms. Larger black dots indicate outliers.

Regarding pathogen copy number, the botanical origin of honey significantly influenced CBPV (Kruskal-Wallis, χ^2^ = 123.11; df = 36; p < 0.001) and ABPV (Kruskal-Wallis, χ^2^ = 65.992; df = 36; p < 0.01). CBPV copy number in acacia, citrus, and strawberry tree honeys was significantly higher compared to lavender, coriander, and eucalyptus honeys. Additionally, asphodel and heather honeys showed higher loads than eucalyptus honey, and the CBPV copy number in asphodel honey was also significantly higher than in lavender honey. Citrus honey displayed a statistically higher CBPV copy number compared to chestnut, linden, multifloral, and rhododendron (*Rhododendron* L.) honeys (Fig 2 and S4 Table). ABPV copy number was significantly higher in reynoutria and teucrium honeys compared to 29 and 30 other honey types, respectively (S4 Table).

**Fig 3:**
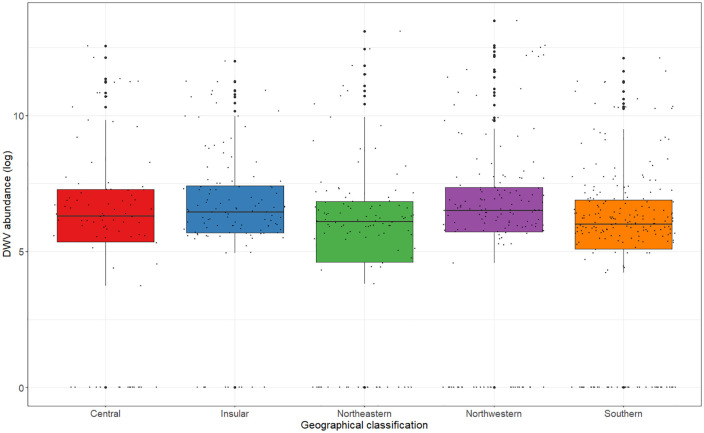
Boxplot of DWV copy number in different geographical areas of Italy. Copy numbers are represented as decimal logarithms. Larger black dots indicate outliers.

The region of honey production influenced *L. passim* copy number (Kruskal-Wallis, χ^2^ = 33.25; df = 19; p < 0.05). Similar to prevalence, the pathogen load was significantly higher in honey produced in Tuscany compared to Abruzzo (S4 Table). DWV copy number was associated with geographical origin (Kruskal-Wallis, χ^2^ = 14.52; df = 4; p < 0.01), with southern Italy showing significantly lower virus loads than the insular and northwestern regions (Fig 3 and S4 Table).

**Fig 4:**
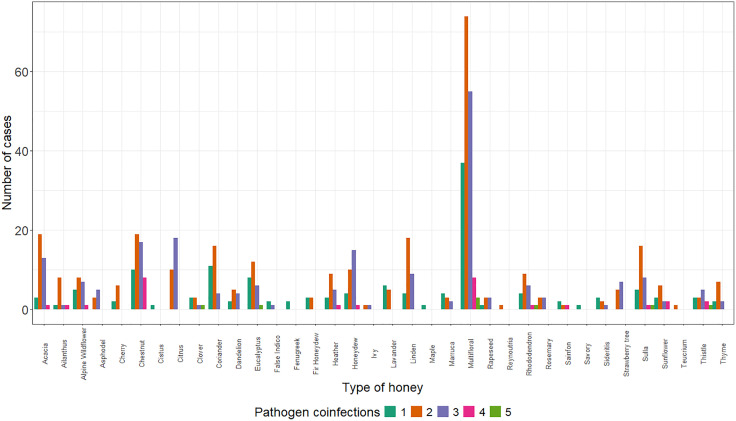
The number of cases in which the investigated pathogens were involved in multiple presences associated with different botanical origins of honey.

A total of 526 honey samples (77.47%) from 32 out of 36 analysed honey types (botanical origins) were found to be co-infected with two or more pathogens (Fig 4). Co-presence of three pathogens was detected in 26 honey types, while co-presence of four pathogens was found in 12 honey types. Eight honey samples exhibited the simultaneous co-presence of five pathogens. Specifically, co-presence of DWV, CBPV, ABPV, BQCV, and *N. ceranae* was found in one French honeysuckle honey sample, one rhododendron honey sample, one eucalyptus honey sample, and two multifloral honey samples. Additionally, one thistle honey sample, one clover honey sample, and one multifloral honey sample were co-infected with DWV, CBPV, BQCV, *N. ceranae*, and *L. passim*.

## Discussion

Honey is the most important beekeeping product, but it is also a valuable resource to accurately monitor the internal conditions of the hive. In fact, honey can contain traces of DNA and RNA of various organisms, acquired through contact with various matrices within the hive [9,32,50,51], including pathogens [23,25,26,28–30,52]. The results of this study show that DNA and RNA from pathogens are present in the honey collected and packaged by beekeepers. This confirms that major pathogens can be detected in honey, as previously demonstrated [11,29,32,52] and that viruses remain detectable even after the extraction and jarring processes if the honey is not heat-treated. This opens a new frontier in the non-invasive monitoring of colony health, although potential contamination from unclean equipment cannot be ruled out. Moreover, the sampled honey was a blend from multiple colonies, so further studies should focus on the direct relationship between honey and the colonies that produced it.

The pathogens detected in honey samples from across Italy reflect the epidemiological situation of colony health in *A. mellifera* populations [33,41,53–55]. DWV, CBPV, and *N. ceranae* are the most prevalent pathogens, while ABPV, BQCV, KBV, and trypanosomatids were found to be less common. The observed correlation between DWV and ABPV may be linked to their transmission routes. Within the colony, the spread of both DWV and ABPV is exacerbated by the ectoparasitic mite *V. destructor* [56–58]. Therefore, the high presence of these pathogens in honey could also serve as an indicator of *Varroa* infestation levels in the originating colony.

Considering the prevalence and copy number of the detected pathogens, the type and region of honey production showed distinct trends for specific pathogens. These differences may be influenced by various factors, including the seasonal pathogen dynamics, the flowering of specific plant species, and migratory beekeeping practices to follow bloom periods, particularly for monofloral honeys. The dynamics of pathogens are closely linked to plant flowering, as foraging on flowers represents one of the main transmission routes, not only among honey bees but also among other pollinators [42,59–62]. It is well known that many pathogens follow the development of the colony, with higher prevalence or abundance at specific times of the year, especially related to the brood cycles [63–66]. The spread of certain pathogens during nectar collection in these periods could be reflected in the honey produced. Honey bees, being oligolectic and generalist foragers, collect nectar and pollen from a wide variety of flowers, following abundant bloom cycles [67,68]. Plant phenology, with overlapping flowering periods of different species, may attract foragers differently, which could explain the variations observed in different honey types. Additionally, migratory beekeeping for pollination services and monofloral honey production may contribute to the wider environmental spread of certain pathogens [13,34,69–71].

The geographical distribution of pathogens in the analysed honey samples revealed a correlation between the presence of DWV in southern Italy compared to other regions. These differences may be linked to variations in apiary health management among beekeepers, potentially influenced by different traditions or practices [72–74]. The climatic conditions of the larger Italian islands may create a more favourable environment for DWV infections [33,75–78]. Besides, in the broader context of climate change, rising temperatures could lead to an increased prevalence of pathogens typically found in warmer climates beyond Europe [79–81].

The presence of *L. passim* in honey is a particularly interesting finding. Currently, this trypanosomatid appears to be relatively uncommon in Italy, with unclear seasonal patterns. The results of this study confirm that honey serves as a valuable sampling matrix for assessing the spread of this protozoan, as previously demonstrated [25,33,34,55,82,83]. Regional abundance levels detected in honey are higher than those identified in forager bees monitored across the country [84], suggesting a wider distribution of the pathogen. Many honey samples showed the co-presence of multiple pathogens, particularly in multifloral honey. This aligns with the condition of honey bee colonies, where multiple pathogens often coexist [85–90]. Multifloral honey is derived from nectar collected from various botanical species, and as bees forage on different plants, they may come into contact with a wide range of pathogens [13,34,91–95].

In this study, primers specifically designed for the RNA and DNA of bee pathogens were used. The findings also raise important considerations regarding the practical implications of honey-based pathogen surveillance. The detection of DNA in honey may reflect past exposure events, as genetic material can persist for long periods within this matrix, whereas RNA is typically more labile and therefore more indicative of recent or ongoing pathogen activity. Consequently, while honey eDNA/eRNA analysis provides a powerful tool for mapping pathogen circulation at a population level, it should not be interpreted as direct evidence of clinical disease within individual colonies. Rather, it complements colony-level diagnostics by offering large-scale epidemiological insights into the distribution of infectious agents. Additionally, honey’s antimicrobial properties may inactivate, degrade and/or destroy the genetic material of pathogens that lack protective resistance mechanisms [96–100]. Nonetheless, the presence of pathogen RNA and DNA in honey indicates direct interaction with bees, their nests, or the flowers they visit, emphasizing the need for appropriate management strategies. Beekeeping practices that involve feeding colonies with honey, such as the transfer of combs from one colony to another, may thus represent a real risk of pathogen spread, facilitating horizontal pathogen transfer, as honey can serve as a reservoir for infectious material [101]. In this context, preventive management strategies and the Good Beekeeping Practices (GBPs) are essential to reduce the spread of pathogens within colonies [102,103]. Pathogen pressure is known to reduce colony productivity, increase winter mortality, and necessitate more frequent treatments [35,104,105]. These factors may lead to reduced honey yields and greater expenditures for colony replacement, ultimately impacting the economic sustainability of beekeeping. Surveillance tools that allow for the early identification of infection hotspots may therefore provide an important contribution to mitigating economic losses and improving long-term resilience in the sector.

Finally, although the detection of pathogen RNA and DNA in honey does not confirm the viability or infectivity of the microorganisms, some agents such as *N. ceranae* spores or viruses associated with *V. destructor* can remain infectious in hive products. The possibility that honey serves as a transmission route within or between colonies cannot be excluded, particularly through trophallaxis or colony feeding. This dual role of honey, as both an epidemiological indicator and a potential infection pathway, highlights the need for cautious interpretation of molecular results and reinforces the importance of adopting biosecure management practices in beekeeping.

## Conclusion

This study demonstrates that honey is a valuable non-invasive matrix for detecting honey bee pathogens through environmental RNA and DNA analysis. The high prevalence of viral and parasitic infections detected in honey samples across Italy underscores its potential as a surveillance tool for colony health. Overall, the molecular detection of bee pathogens in honey provides a fast and effective screening method to assess apiary health. The ability to monitor pathogen presence without disturbing the hive offers a practical and efficient alternative to traditional sampling methods. Further investigations are necessary to determine whether the presence of pathogen RNA and DNA in honey corresponds to actual infection in hives and whether honey analysis can reliably estimate pathogen loads in apiaries, considering that pathogen levels influence disease manifestation.

## Supporting information

S1 Table Details and pathogen copies in the investigated honey samples. (XLSX)

S2 Table Relationship between Principal Components (PC1 and PC2) and pathogen copy number variables. (DOCX)

S3 Table P-value correlation between pathogens. (DOCX)

S4 Table Results of the comparative statistical test for the prevalence and abundance of the analysed pathogens by type of honey, region and geographical classification. (XLSX)

S1 Fig a) Prevalence (top graph) and abundance (bottom graph) per type of honey for the investigated pathogens; b) Prevalence and abundance per region for the investigated pathogens; c) Prevalence and abundance per geographical classification for the investigated pathogens. Prevalence (in blue) is shown as a percentage, while abundance (in red) is shown as a decimal logarithm.
(DOCX)
