# Supplementary material for: Molecular detection of bee pathogens in honey from various botanical origins
Source: PLoS One. 2025 Dec 10;20(12):e0336324. doi: 10.1371/journal.pone.0336324 (PMC12694878; doi:10.1371/journal.pone.0336324)
Supplement: S2 Table — (DOCX) [file pone.0336324.s002.docx]

**Table S2.** Relationship between Principal Components (PC1 and PC2) and pathogen copy number variables.

| **Pathogen** | **PC1** | **PC2** |
| --- | --- | --- |
| DWV | -0.998069820 | -0.012290268 |
| CBPV | 0.011866087 | -0.988368899 |
| ABPV | -0.024189599 | 0.007827622 |
| BQCV | -0.040226890 | -0.001456646 |
| *N. ceranae* | -0.002036081 | 0.151346960 |
| *L. passim* | 0.038837418 | -0.002563735 |
| Variance explained | 41.62% | 25.60% |
