# Supplementary material for: Molecular detection of bee pathogens in honey from various botanical origins
Source: PLoS One. 2025 Dec 10;20(12):e0336324. doi: 10.1371/journal.pone.0336324 (PMC12694878; doi:10.1371/journal.pone.0336324)
Supplement: S3 Table — (DOCX) [file pone.0336324.s003.docx]

**Table S3.** P-value correlation between pathogens.

|  | **DWV** | **CBPV** | **ABPV** | **BQCV** | ***N. ceranae*** | ***L. passim*** |
| --- | --- | --- | --- | --- | --- | --- |
| **DWV** |  | 0,173 | 0,002 | 0,000 | 0,123 | 0,166 |
| **CBPV** | 0,173 |  | 0,175 | 0,983 | 0,100 | 0,909 |
| **ABPV** | 0,002 | 0,175 |  | 0,059 | 0,164 | 0,694 |
| **BQCV** | 0,000 | 0,983 | 0,059 |  | 0,521 | 0,722 |
| ***N. ceranae*** | 0,123 | 0,100 | 0,164 | 0,521 |  | 0,672 |
| ***L. passim*** | 0,166 | 0,909 | 0,694 | 0,722 | 0,672 |  |
