# Supplementary material for: Molecular detection of bee pathogens in honey from various botanical origins
Source: PLoS One. 2025 Dec 10;20(12):e0336324. doi: 10.1371/journal.pone.0336324 (PMC12694878; doi:10.1371/journal.pone.0336324)
Supplement: S1 Fig — Prevalence (in blue) is shown as a percentage, while abundance (in red) is shown as a decimal logarithm. (DOCX) [file pone.0336324.s005.docx]

a)


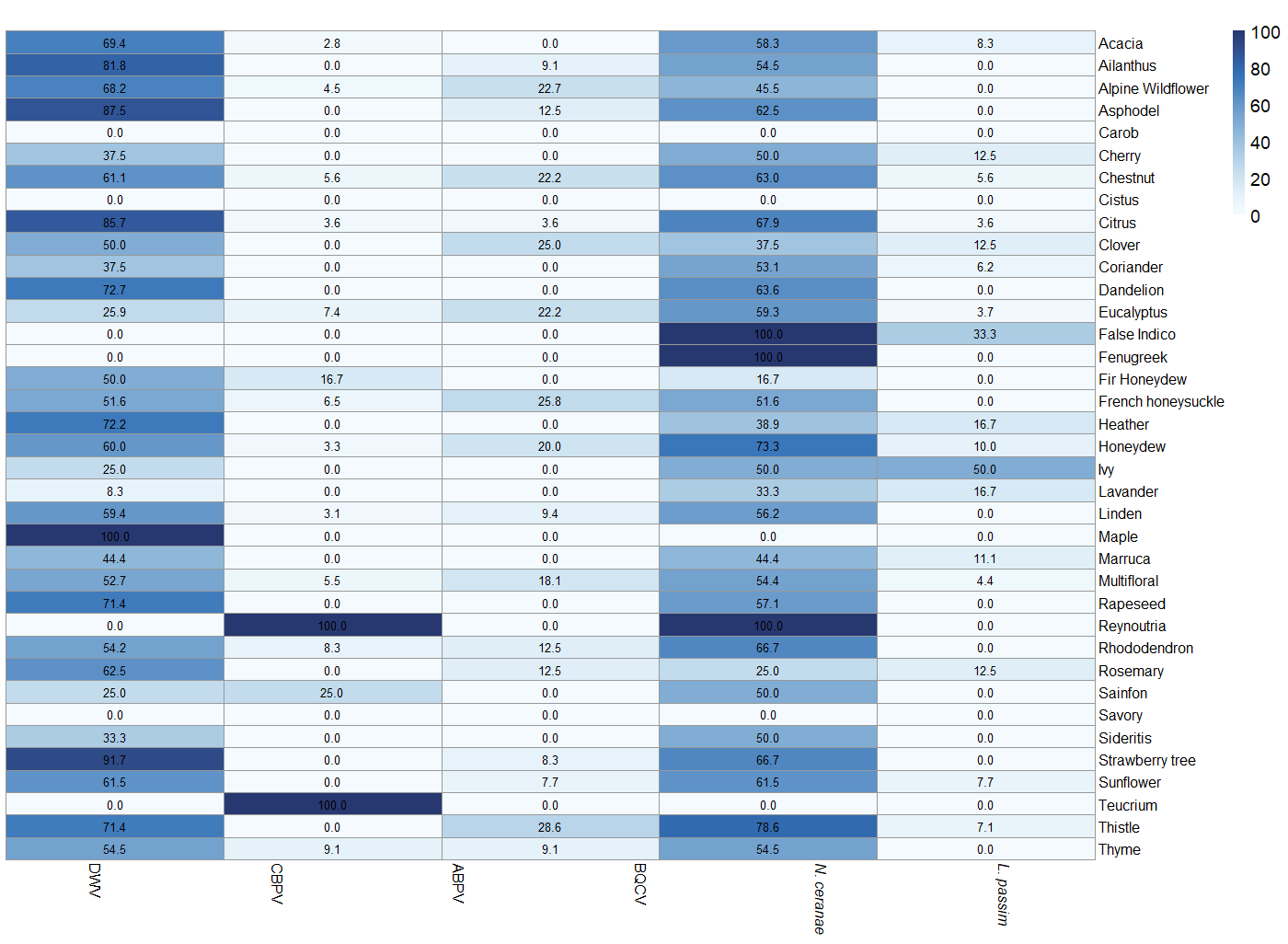

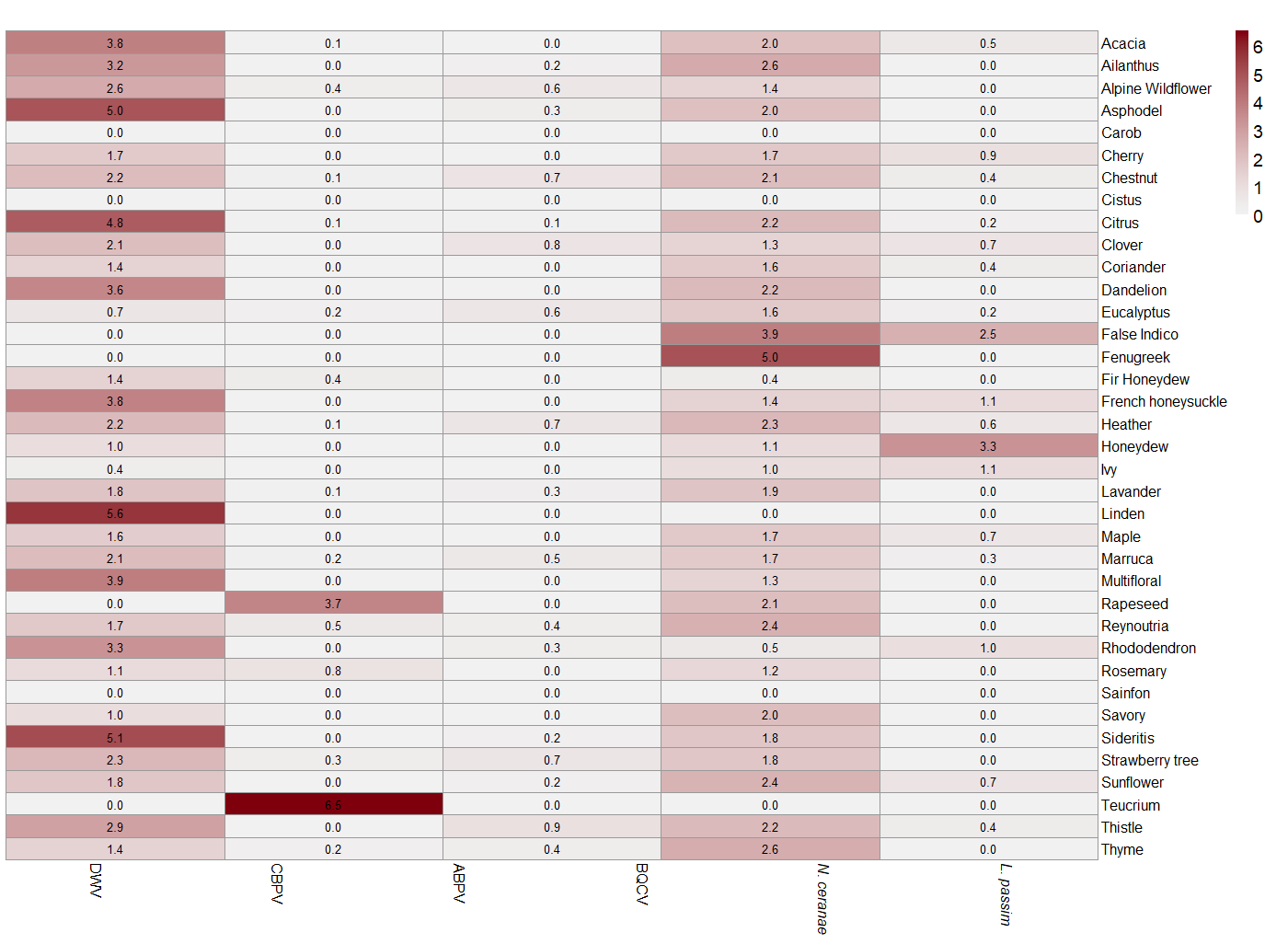


b)


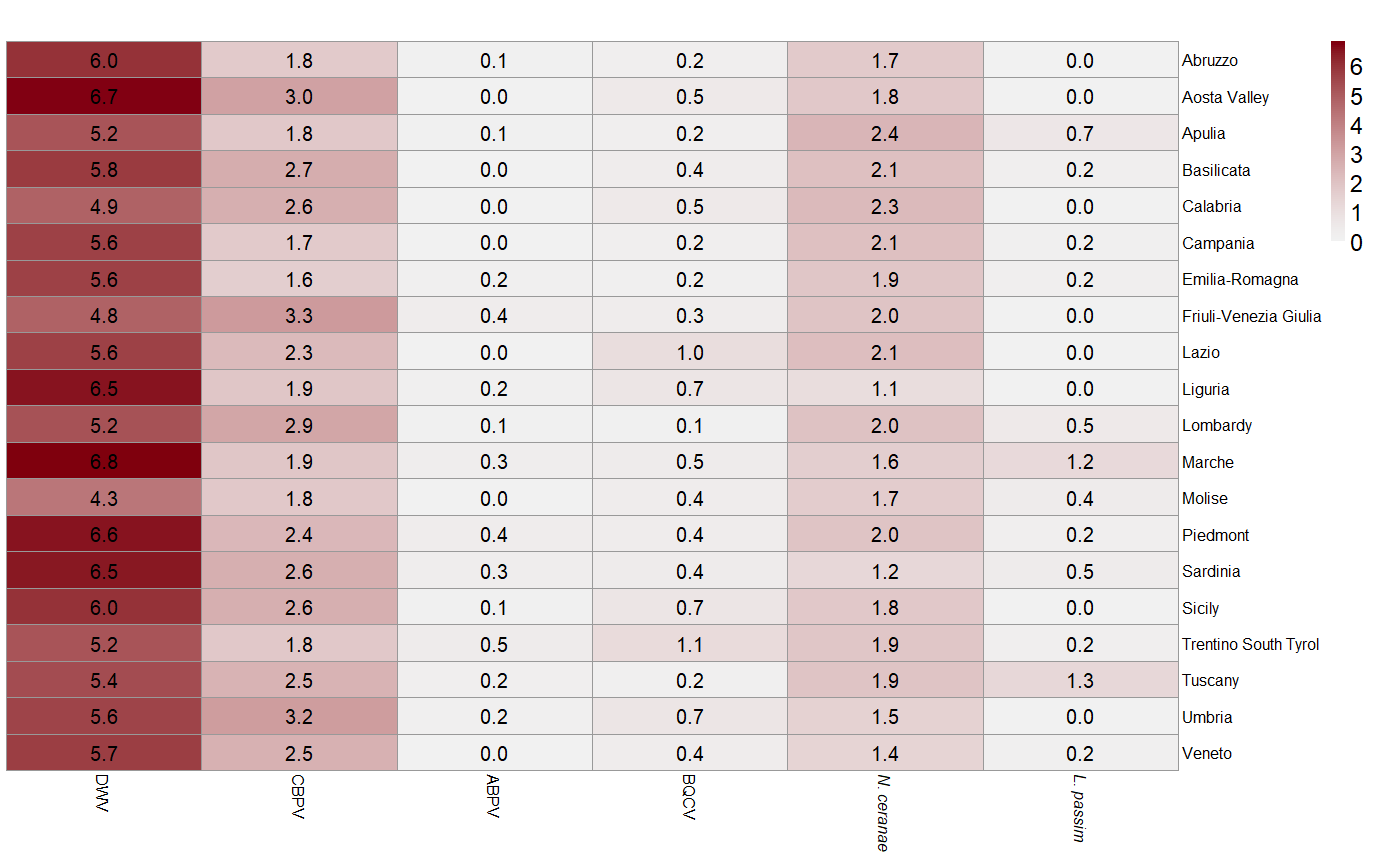

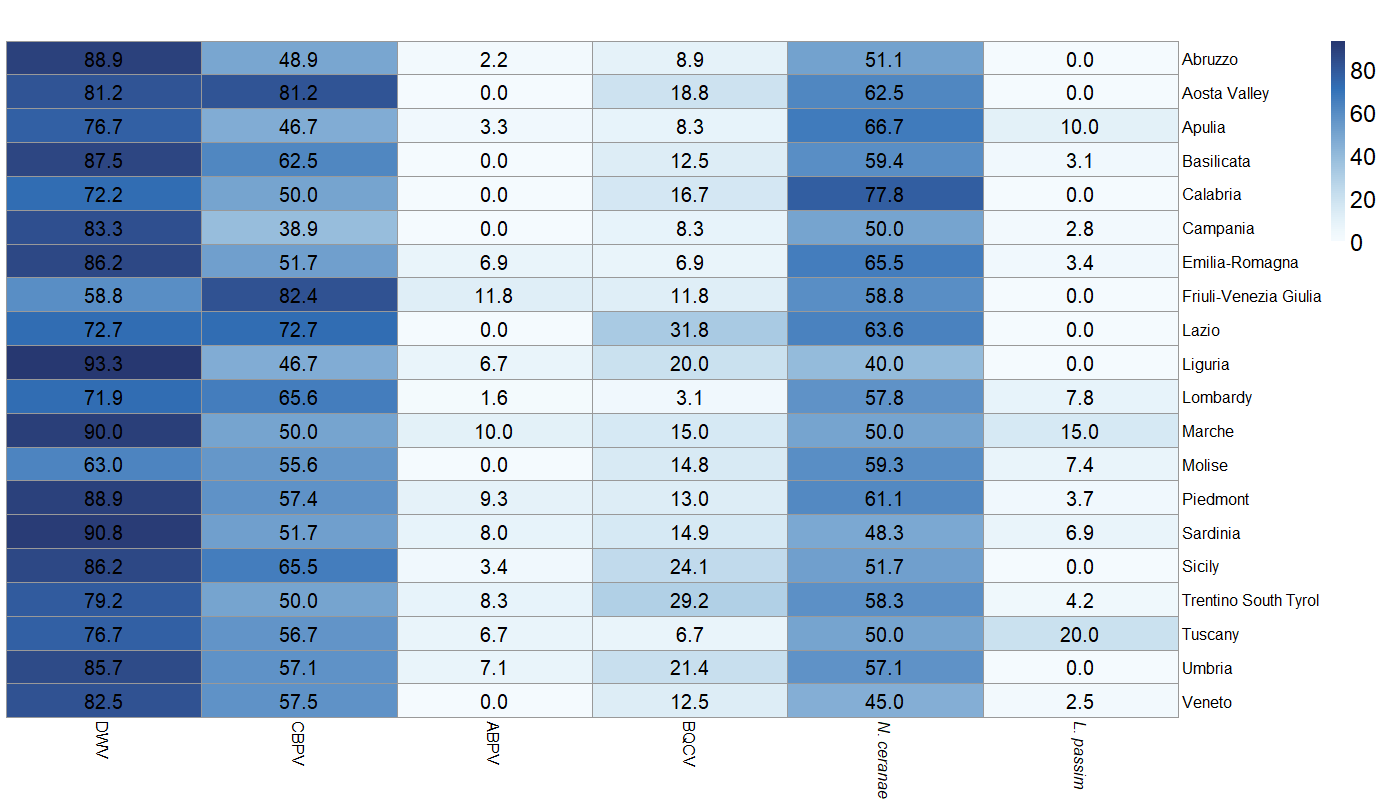


c)


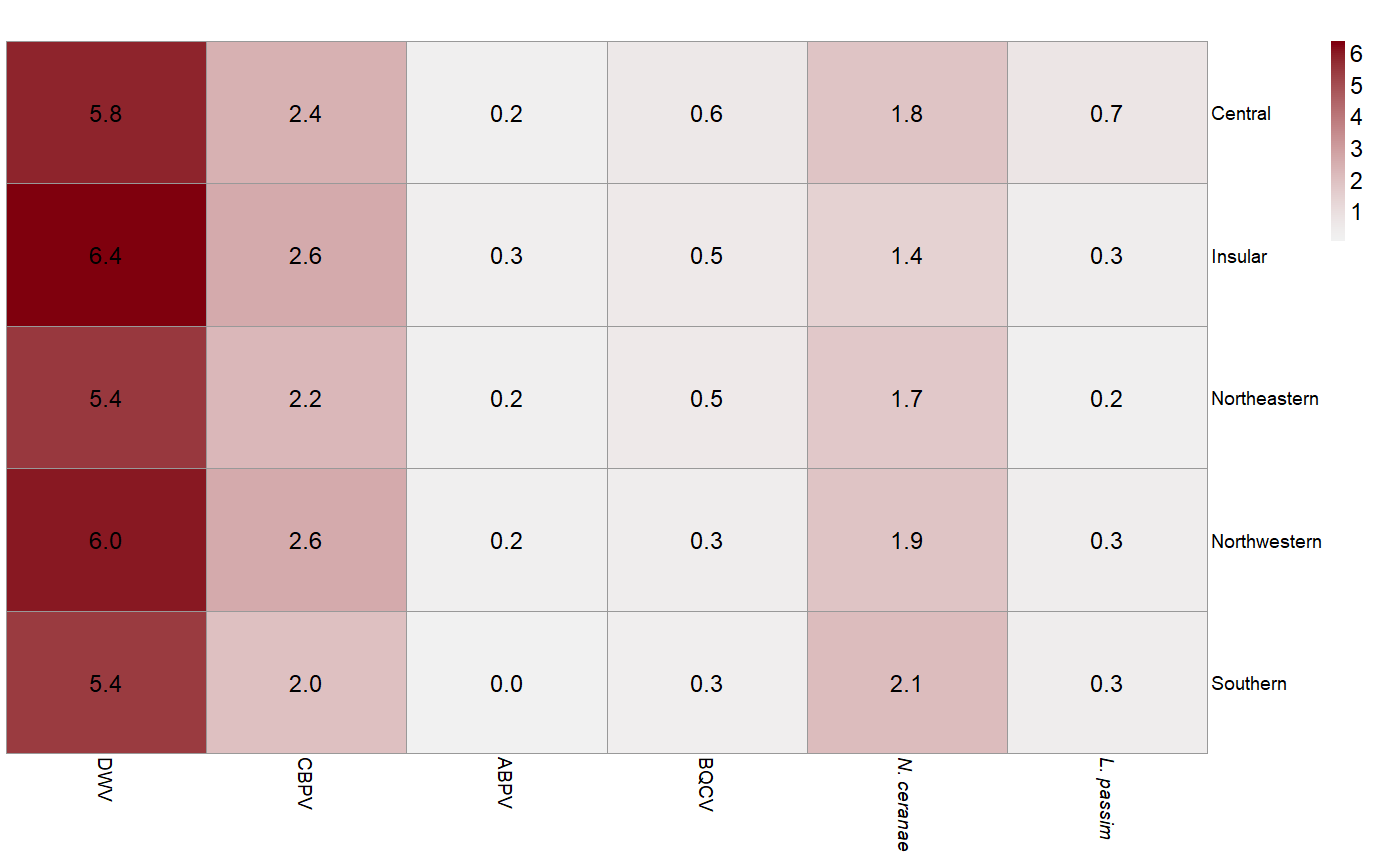

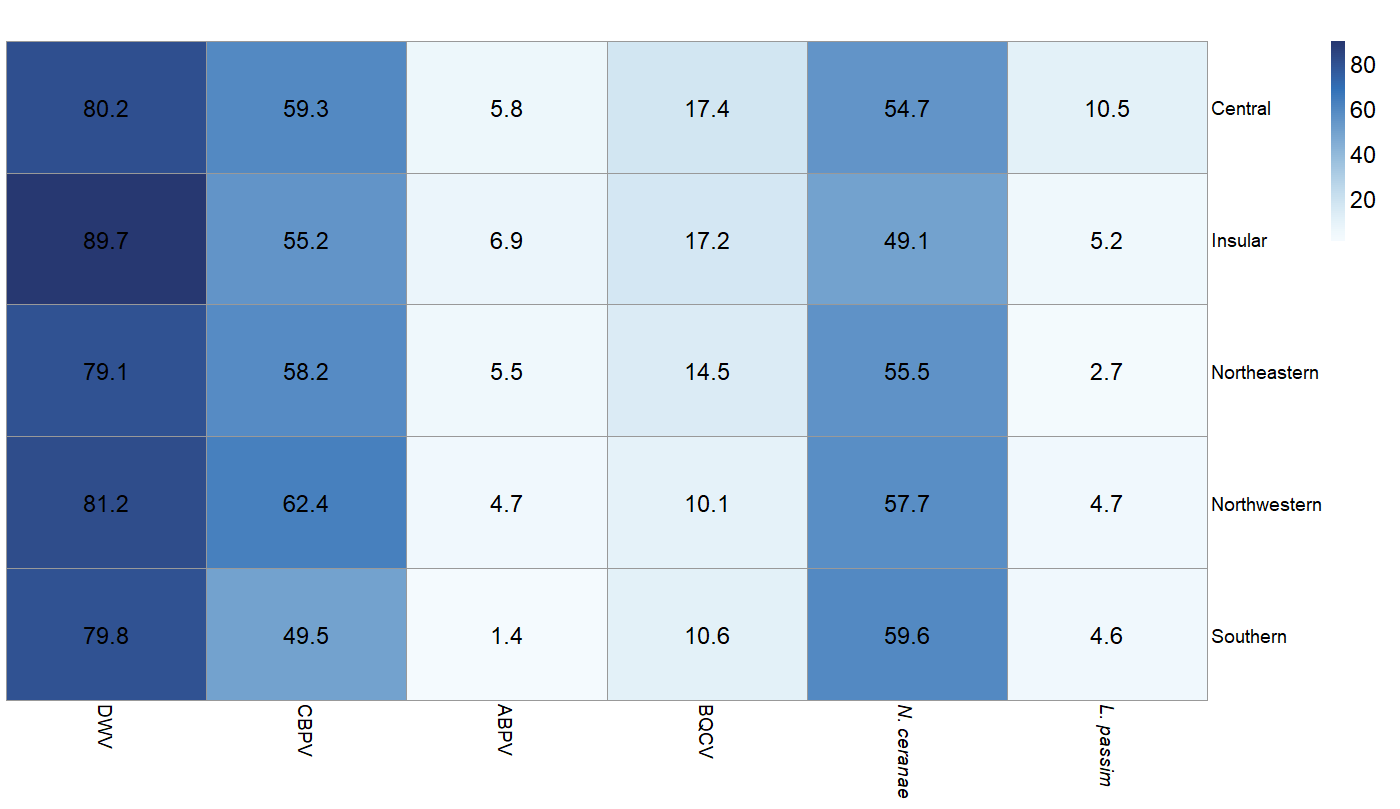


Figure S1. a) Prevalence (top graph) and abundance (bottom graph) per type of honey for the investigated pathogens; b) Prevalence and abundance per region for the investigated pathogens; c) Prevalence and abundance per geographical classification for the investigated pathogens. Prevalence (in blue) is shown as a percentage, while abundance (in red) is shown as a decimal logarithm.
